# Novel prognostication of patients with spinal and pelvic chondrosarcoma using deep survival neural networks

**DOI:** 10.1186/s12911-019-1008-4

**Published:** 2020-01-06

**Authors:** Sung Mo Ryu, Sung Wook Seo, Sun-Ho Lee

**Affiliations:** 1 Department of Neurosurgery, Spine tumor center and Special Cancer center, Samsung Medical Center, Sungkyunkwan University School of Medicine, 81 Irwon-ro, Gangnam-gu, Seoul, 06351 South Korea; 2 Department of Orthopedic Surgery, Special Cancer center, Samsung Medical Center, Sungkyunkwan University School of Medicine, 81 Irwon-ro, Gangnam-gu, Seoul, 06351 South Korea

**Keywords:** Artificial intelligence, Chondrosarcoma, Neural network, Prediction, Survival

## Abstract

**Background:**

We used the Surveillance, Epidemiology, and End Results (SEER) database to develop and validate deep survival neural network machine learning (ML) algorithms to predict survival following a spino-pelvic chondrosarcoma diagnosis.

**Methods:**

The SEER 18 registries were used to apply the Risk Estimate Distance Survival Neural Network (RED_SNN) in the model. Our model was evaluated at each time window with receiver operating characteristic curves and areas under the curves (AUCs), as was the concordance index (c-index).

**Results:**

The subjects (*n* = 1088) were separated into training (80%, *n* = 870) and test sets (20%, *n* = 218). The training data were randomly sorted into training and validation sets using 5-fold cross validation. The median c-index of the five validation sets was 0.84 (95% confidence interval 0.79–0.87). The median AUC of the five validation subsets was 0.84. This model was evaluated with the previously separated test set. The c-index was 0.82 and the mean AUC of the 30 different time windows was 0.85 (standard deviation 0.02). According to the estimated survival probability (by 62 months), we divided the test group into five subgroups. The survival curves of the subgroups showed statistically significant separation (*p* < 0.001).

**Conclusions:**

This study is the first to analyze population-level data using artificial neural network ML algorithms for the role and outcomes of surgical resection and radiation therapy in spino-pelvic chondrosarcoma.

## Introduction

The Surveillance, Epidemiology, and End Results (SEER) database has been queried in a series of reports to analyze all primary malignant tumors of the osseous spine, including chondrosarcoma [1–4]. The SEER registry has been collecting cancer-related information since 1973, and it represents 28% of the total U.S. population today, serving as the only population-based comprehensive data source, including stage of cancer, treatment modality and survival data [5]. However, most of previous studies included in the SEER database have either included only a basic demographic description or have excluded patients with incomplete data in multivariable analysis [6–8].

Machine learning (ML) provides the opportunity to analyze heterogeneous and complex data due to the greater capability to identify unintuitive patterns in large patient datasets [9]. Several ML algorithms have been applied in clinical medicine to predict disease, and they have shown a higher accuracy in diagnosis when compared to classical methods [10]. ML models have been expected to be useful for small datasets typical of rare pathologies such as primary bone tumor and showed the good discrimination and performance on decision analysis [11].

In the present study, we hypothesized that applying ML techniques may be equally valuable in other clinical areas, such as in identifying the prognostic factors in spinal and pelvic chondrosarcoma. We seek to develop and validate recurrent neural network ML algorithms to precisely predict survival following a diagnosis of chondrosarcoma using a national database.

## Methods

### Data collection

The SEER database is a longitudinal database that collects information from 17 population-based cancer registries (http://seer.cancer.gov/). Serial registry data are de-identified and submitted to the United States National Cancer Institute on a biannual basis to make available to researchers. The primary data in the SEER database includes the following variables: age at diagnosis, gender, race, primary site and size of tumor, histology, grade of tumor, stage of tumor, surgical treatment, radiation and chemotherapy, and overall survival (OS) in months. The original data set has 193 variables. (https://seer.cancer.gov/data-software/documentation/seerstat/nov2016/).

In this study, patients with chondrosarcoma diagnosed from 1973 to 2014 were selected using the Histologic International Classification of Diseases for Oncology, Third Edition (ICD-O-3), codes 9220–9243. The sites of presentation were compiled according to ICD-O-3 topography codes and were grouped into vertebral and pelvic sites in a fashion similar to that in previous studies [6, 8]. The extent of tumor was reclassified based on SEER EOD (Extent of Disease) and CS (Collaborative Stage) into three groups: ‘confined’ (defined as tumor encasement within the periosteum), ‘locally invasive’ (beyond the periosteum without distant involvement), and ‘distant’. Cases only with an autopsy/death certificate were excluded due to the unknown survival periods. As the SEER database uses publicly available data without personal identifiers, an approval from Institutional review board and/or informed consent are not required.

### Study data preparation

**Fig. 1:**
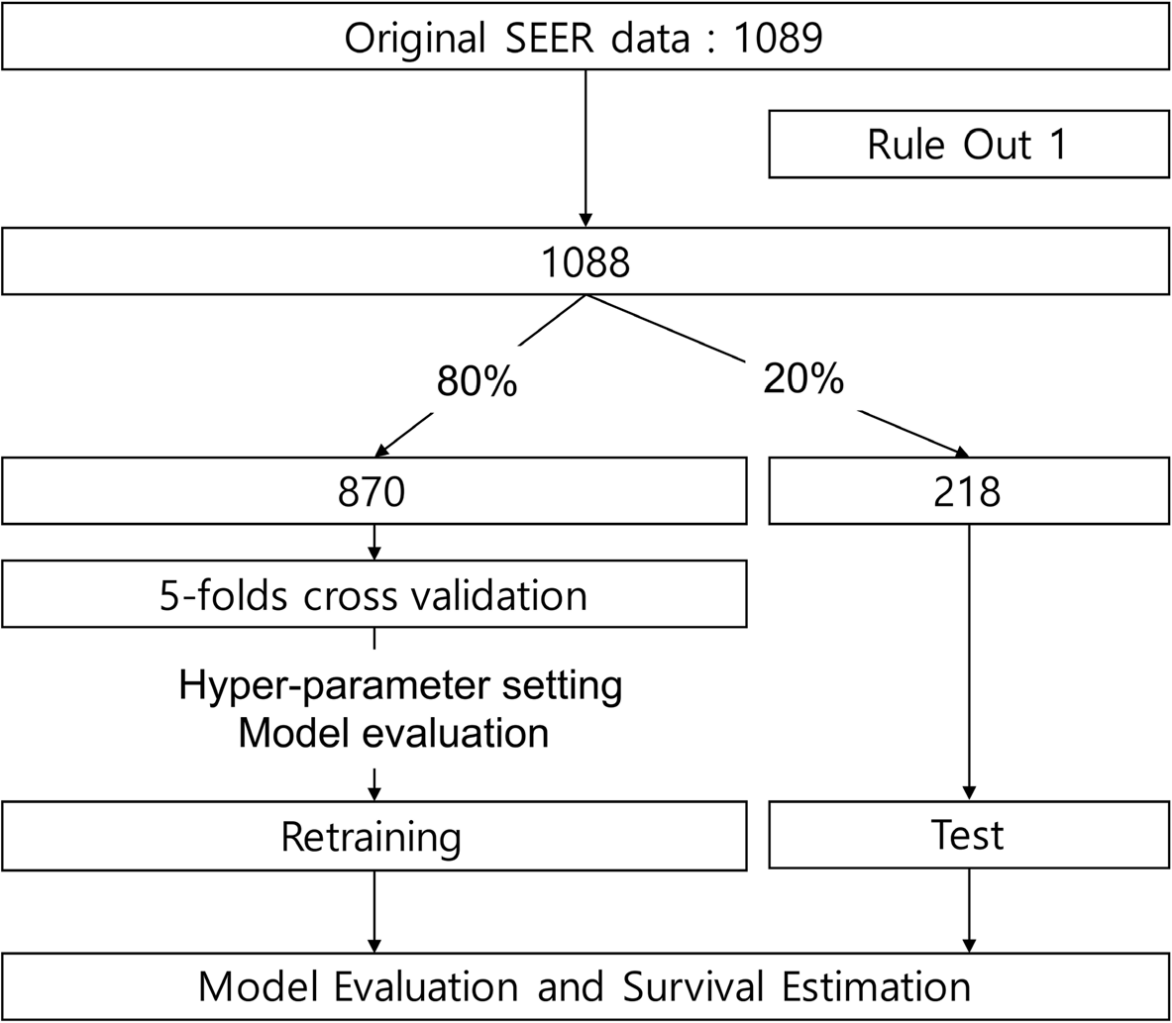
Enrolled Study Population and Pipeline of Data Analysis. The training data were validated using 5-fold cross validation

The fourteen input variables for the study include sex, ethnicity (white, black, Hispanic, or other), age at diagnosis, marital status, primary site (spine and pelvis), tumor size, histologic type, grade, laterality, SEER historic stage, surgery, radical resection, radiation, chemotherapy, and total number of in situ*/*malignant tumors for the patient. The patient’s age at diagnosis was grouped into 3 categorical variables with intervals of 30 years. The tumor size was divided into two groups (> 8 or ≤ 8 cm) according to the T stage classification for bone tumors determined by the American Joint Committee on Cancer. The age, grade, tumor size, and number of tumors were used as discrete ordinal values. The other variables were considered to be categorical. All subjects (*n* = 1088) were separated into a training set (80%, *n* = 870) and a test set (20%, *n* = 218). The training data were randomly sorted into the training set and validation set using 5-fold cross validation (Fig. 1). This step was repeated to optimize the hyperparameters. Adjusting the hyperparameters, the median values of the c-index among the five tests were evaluated to find the optimal hyperparameters (number of learning epochs, risk value, and time window). After the optimization step, the final algorithm was retrained with the whole training set and evaluated with the test set. After separating the training and test sets, the missing values of the input variables were imputed using the k-nearest neighbor algorithm and transformed using Standard Scaler.

### Risk estimate distance survival neural network (RED_SNN)

**Fig. 2:**
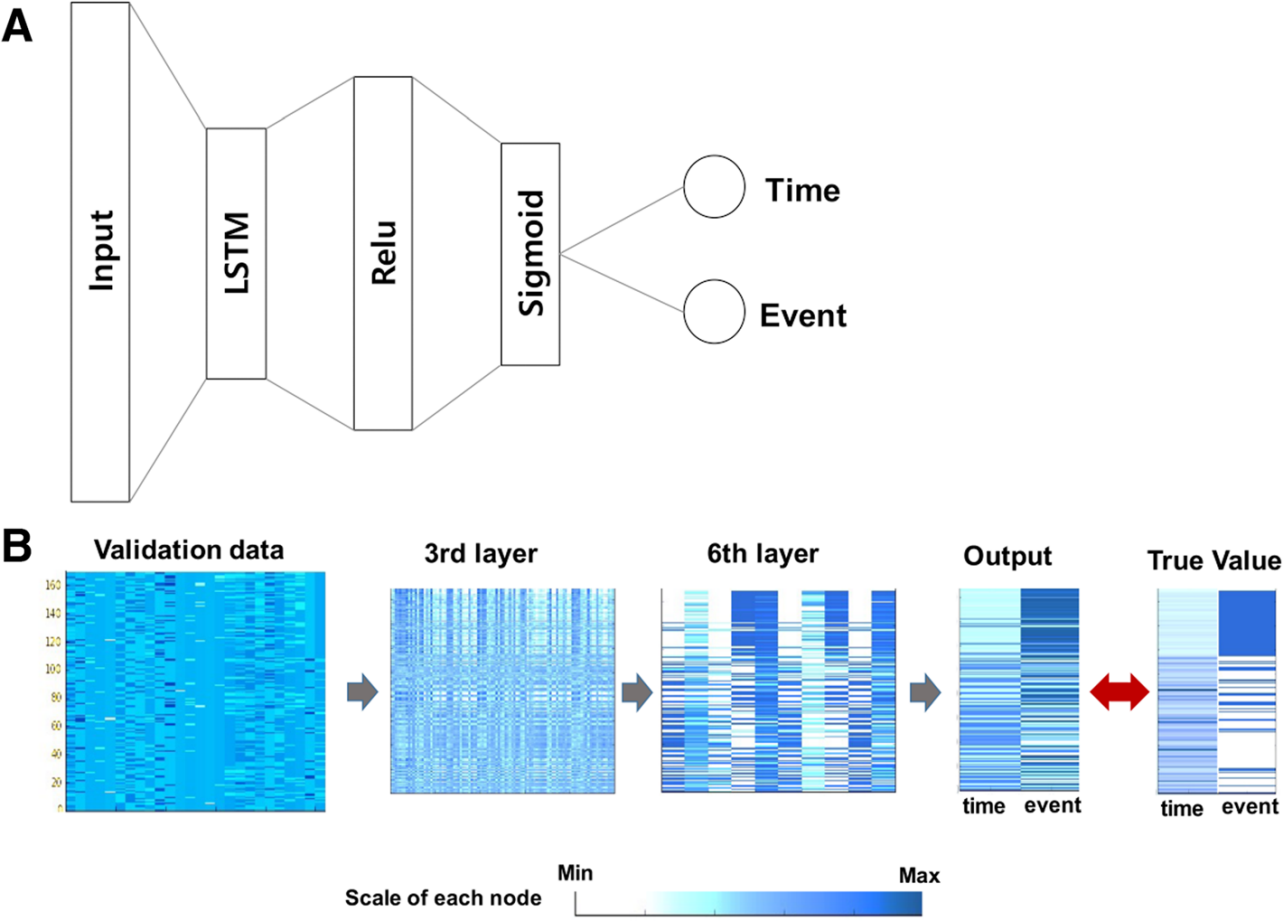
The architecture of the basic learning unit of the RED_SNN model. (**a**) The network architecture of the basic unit was composed of 8 layers, including two long short term memory (LSTM) layers. The input layer was comprised of 28 nodes that represented 26 input features and 2 latent survival features. The output layer was composed of 2 nodes implementing linear function, representing time and event. Since the two target nodes have different characteristics, we did not use the softmax function. The other layers were composed of fully-connected nodes implementing a rectified linear unit function. (**b**) The validation data (*n* = 169) were inputted to the pre-trained network. The number of nodes was gradually reduced across the hidden layers. The output time and event were compared to the true target values

The key point of this model is that the event and time should be located in different dimensions, and the neural network learns these two targets at the same time using a multimodal algorithm (Fig. 2, Table 1, Additional file 1: Figure S1 and Additional file 2: Table S1). The event is defined as a binary value (survive = 0 or death = 1) in the time window period, which requires a logistic model. Time is the total follow-up period of each patient defined as a continuous variable (month), which requires a regression model. Our multimodal algorithm enables learning those two different characters by estimating the risk estimate distance (RED). In the time window period, subjects were located in the time dimension and the survival dimension. In the survival dimension, the event cases will have a risk score (α) that can be specified by the severity of each cancer. In this vector space, RED is defined as the cosine distance between survival and death in a certain time window. Thus, RED is higher in cases of earlier death, which represents a higher risk.

$$ {\displaystyle \begin{array}{c}\boldsymbol{Risk}\ \boldsymbol{Estimate}\ \boldsymbol{Distance}\ \left(\boldsymbol{RED}\right)= cosine\ distance\\ {}=1-\mathit{\cos}\varnothing =1-\frac{A\bullet B}{\left|A\right|\left|B\right|}\end{array}} $$

**Table 1:**
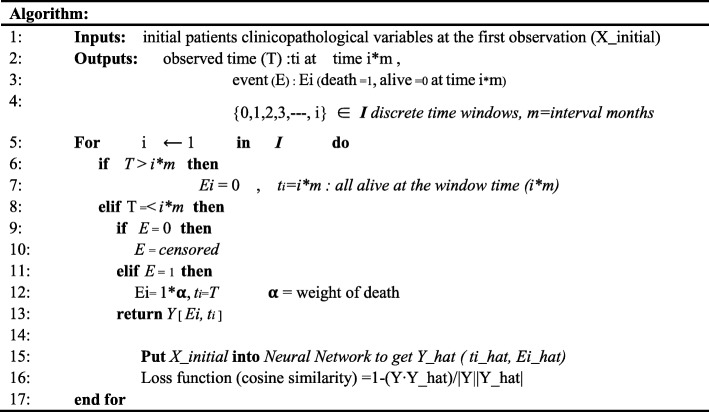
Algorithm of risk estimate distance survival neural network

X_initial is observed variables of each patients at the first visit. Targets include time and event. Time target (T) is a continuous variable representing follow-up months

m is the time window or the interval time, which is a tunable hyper-parameter (In this study, the interval time was 10 month)

ti is the last observed time during the time interval i

Survival target (E) is a binary value representing event (alive: 0, death: 1). Ei is a binary event during the time interval i. The neural network is trained with the targets Y[Ei, ti] recurrently at each time point i using cosine distance as a loss function. (훂 is a hyperparameter representing weight of death.) For example, E1 = 0 at the first time window could be E2 = 0, 1, or censored at the second window, thus the neural network should adjust their parameter to the following targets. After their serial training, the network learned to perceive severity of the cancer patient

The parameter of the model (θ) should be inferred using the cosine proximity loss function (${\mathcal{L}}_{\left(\theta \right)}$**)** as follows (X_i_: features of i-th subject, n: subjects).

Y_hat (time_i_, event_i_) = g (θ; X_i_), {1, 2, …*i*} ∈ *n*

$$ {\mathcal{L}}_{\left(\theta \right)}=1- cos\theta =1-\frac{Y\bullet Y\_ hat}{\left|Y\right|\left|Y\_ hat\right|} $$

where Y_hat is the estimated outputs, Y is the observed time and event.

In the next time period, some patients will die (new event) and some will be lost (censored), thus the observed time and event will change in the next time window. The algorithm is trained serially with the different target value at each time window, and the model parameter (θ) will be gradually adjusted to the time serial targets. (In this algorithm, we did not consider the change in the features (X) at each time window because our algorithm is designed to estimate the prognosis with the first status of the patients.) The algorithm was trained using backpropagation and was optimized using RMSProp at every two months (time window (m) = 2) with a single epoch and risk score (α) = 10.

### Model evaluation and statistical analysis

The present study was developed and written according to transparent reporting of a multivariable-prediction model for individual prognosis or diagnosis model development guidelines. Patient outcomes were measured based on OS, the period (in months) between diagnosis and death or loss of follow-up from any cause, as reported in the SEER database. The performance of our prediction model was evaluated at each time window with the receiver operating characteristic (ROC) curves and areas under the curves (AUCs). Using the last follow-up information, the concordance index (c-index) of the model was also evaluated. The AUCs and c-indexes were compared between the groups using the Mann-Whitney test. The calibration curves were estimated by comparing the mean probability of the predicted survival with the actual survival proportion using Kaplan-Meier estimates after subgrouping of the test group into seven equally numerous subsets.

In the test group, the SEER historic stage and the subgroups classified by the predicted survival probability were compared using Kaplan-Meier curves. Each curve was compared to the neighboring curves using the log-rank test. Comparisons between groups were considered to be statistically significant at *p* < 0.05. We used the Keras and Theano libraries to obtain the deep learning framework. Data preprocessing was performed using the scikit-learn library. Statistical analysis was performed with SPSS version 24.0 (SPSS Inc., Chicago, IL, USA).

## Results

### Patient demographics

The search identified 1089 patients with primary chondrosarcoma of the osseous spine and pelvis between 1973 and 2014. Only one case was excluded owing to an unknown survival period. Among the remaining 1088 patients, 62.0% were men and 86.2% were white (Table 2). The mean and median age of diagnosis were 51.7 and 52 years, respectively. The year of diagnosis was 2000 or later for 65.3% of cases.

**Table 2:** Patient demographics, tumor characteristics, and treatment modality

	Characteristic	
Age at diagnosis, yrs	Mean ± SD	51.70 ± 18.81
	Median (range)	52.00 (8–93)
Age at diagnosis stratified, n (%)	0–29	
	30–59	
	> 59	
Sex, n (%)	Male	675 (62.0)
	Female	413 (38.0)
Race, n (%)	White	938 (86.2)
	Black	79 (7.3)
	Other (Asian)	59 (5.4)
	Unknown	12 (1.1)
Primary site involved, n (%)	Vertebral column	218 (20)
	Pelvic bones, sacrum	870 (80)
Histology type	Chondrosarcoma, NOS	948 (87.1)
	Juxtacortical chondrosarcoma	7 (0.6)
	Myxoid chondrosarcoma	60 (5.5)
	Mesenchymal chondrosarcoma	23 (2.1)
	Clear cell chondrosarcoma	6 (0.6)
	Dedifferentiated chondrosarcoma	44 (4.0)
Grade	I	300
	II	397
	III	121
	IV	63
	Unknown	207 (19.0)
Stage of the tumor	Localized	411 (37.8)
	Regional	450 (41.4)
	Distant	147 (13.5)
	Unknown	80 (7.4)
Surgery performed, n (%)	Yes	818 (75.2)
	No	249 (22.9)
	Unknown	21 (1.9)
Radiation therapy (%)	Yes	228 (21.0)
	No	848 (77.9)
	Unknown	12 (1.1)
Chemotherapy (%)	Yes	139 (12.8)
	No	949 (87.2)
Cause of death (%)	Tumor related	333 (30.6)
	Other	223 (20.5)
	Censored	532 (48.9)

*NOS* not otherwise specified

Disease was histologically confirmed in 1061 patients (97.5%). In contrast, radiologic and clinical confirmation were performed in 27 patients (2.5%). Among the 1061 patients who received histologic confirmation, most were diagnosed with chondrosarcoma, not otherwise specified (87.1%). Among other histologic types, myxoid (5.5%), dedifferentiated (4.0%), and mesenchymal (2.1%) were common histologic variants. Regarding grade, 64.1% of cases were of low grade (grade I and II), 16.9% were high grade (grade III and VI), and 19.0% were of unknown tumor grade. The mean and median tumor size at the time of diagnosis were 96.6 mm (standard deviation 61.2 mm) and 88.0 mm, excluding 394 patients with unknown tumor size. The extent of the disease was known in 92.6% cases, with the majority presenting as regionally invasive disease (41.4%). The index site was the first malignant tumor; 868 patients had only the index tumor, while 220 patients had two or more tumors.

After diagnosis, 12.3% of patients received both surgery and radiation, 57.9% underwent surgery alone, and 9.0% underwent radiation alone, while 16.2% of patients received neither, and 4.5% had an unknown treatment regimen (Table 3). During follow-up, 556 patients died, and among these, disease-related deaths were confirmed in 333 cases. The survival analysis based on Kaplan-Meier curves revealed that the 5-year OS and disease specific survival for all patients were 55.4 and 69.2%, respectively. The median OS was 92 months.

**Table 3:** Surgical and adjunctive treatment in 1088 patients with spinal and pelvic chondrosarcoma

Surgery (n)	Radiation (n)	Chemotherapy	Number of patients
Yes (818)	No (673)	No	622
		Yes	51
	Yes (135)	No	106
		Yes	29
	Unknown (10)	No	9
		Yes	1
No (249)	No (159)	No	128
		Yes	31
	Yes (88)	No	64
		Yes	24
	Unknown (2)	No	2
Unknown (21)	No (16)	No	14
		Yes	2
	Yes (5)	No	4
		Yes	1

### Training and validation of RED_SNN

**Fig. 3:**
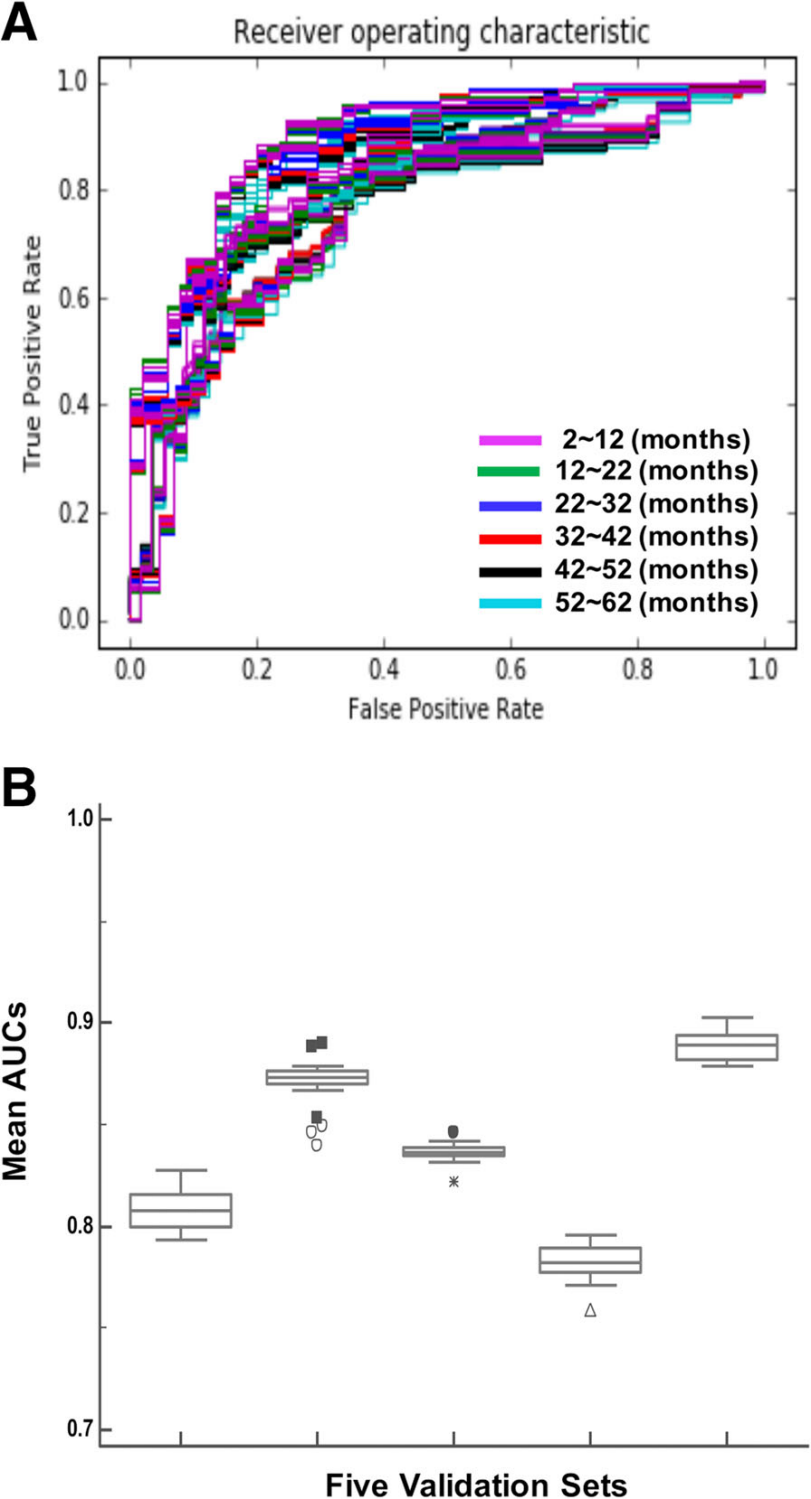
Optimization and validation of RED_SNN model using 5-fold cross validation. **a** ROC curves to evaluate the prediction accuracy of the RED_SNN model. The model was serially trained to learn patient’s survival status within a 10-month time interval, until 62 months from the initial observation. ROC curves predicting survival at each time interval were evaluated with validation sets. **b** The mean AUC’s of the survival prediction at each time point. The average AUC of 5-fold cross validation was 0.84

We optimized the hyperparameters of our model at the risk value: 10, time window: 2 months, and 2 learning epochs. In this setting, the model is trained every two months for 62 months (30 times), with time-dependent target values within a single epoch. The same learning process is repeated with different batches in the second epoch. The median c-index of the five validation sets was 0.84 (95% confidence interval 0.79–0.87). The median AUC of the five validation subsets was 0.84 (Fig. 3a and b).

### Performance evaluation of RED_SNN using test data set

**Fig. 4:**
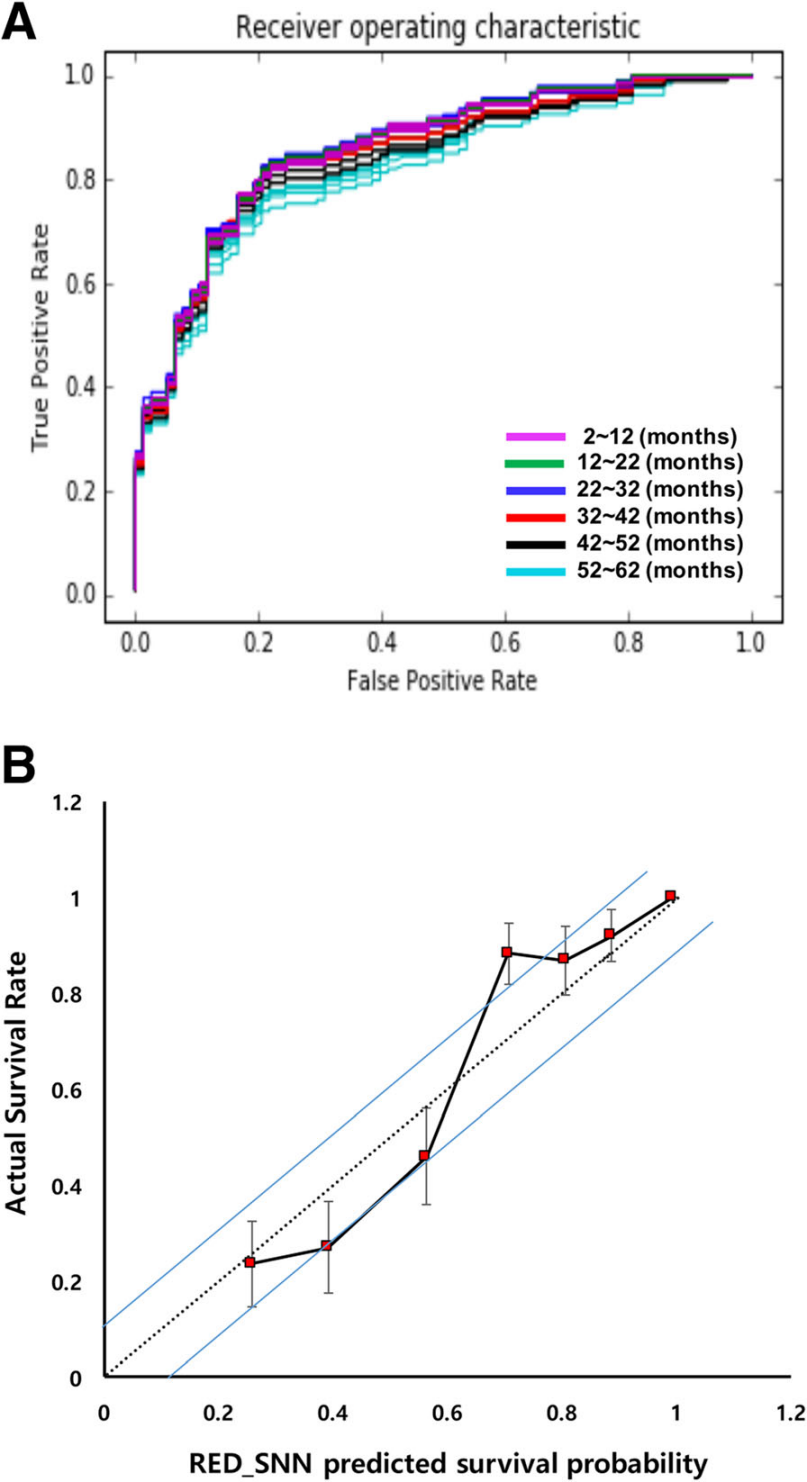
Performance evaluation of RED_SNN using test data set. **a** ROC curves to evaluate the prediction with test data set. The test data were analyzed by pre-trained RED_SNN and its output (expected survival probability) was compared to the real survival of the test set at each time point. **b** Calibration curves of RED_SNN model to predict the survival rate of the test set. The test data were analyzed using pre-trained RED_SNN, and the test patients were equally divided into seven subgroups according to the model’s predicted survival probability at five years

The RED_SNN with fixed hyperparameters (risk value: 10, time window: 2 months, and two learning epochs) was finally trained with the total training set, and the final RED_SNN specified for spinal and pelvic chondrosarcoma was developed. All of fourteen input variables selected initially were applied in final model. This model was evaluated with a previously separated test set. The c-index was 0.82, and the mean AUC of the 30 different time windows was 0.85 (standard deviation 0.02). The calibration curve analysis showed that the predicted survival probability represented the actual survival proportion within a 10% margin of error (Fig. 4a and b).

### Prognostication according to estimated survival probability

**Fig. 5:**
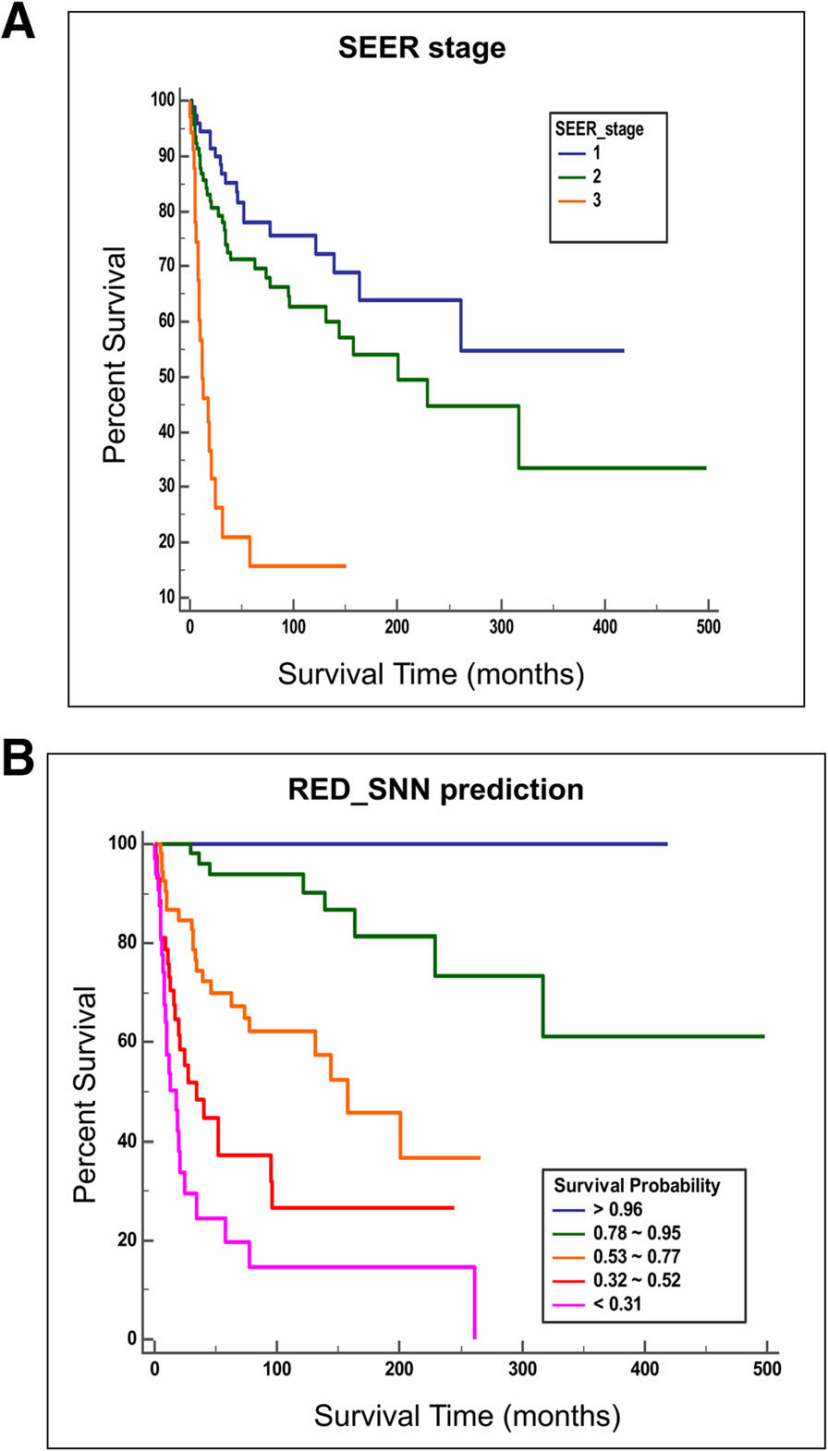
Kaplan Meier curves of subgroups according to SEER stage vs our model expected survival probability. **a** SEER stage identified three prognostic subgroups in Kaplan Meier survival analysis. **b** RED_SNN identified five prognostic subgroups in Kaplan Meier survival analysis

With the test data set, Kaplan-Meier curves were generated according to the SEER historic stages (Fig. 5a). A clear separation of the survival curves was shown for the different stage groups identified using the survival tree method (log-rank test; *p* < 0.001). According to the estimated survival probability (by 62 months), we divided the test group into five subgroups. The first subgroup included patients with estimated survival probability greater than 96% (*n* = 19), and the second subgroup included patients with estimated survival probability between 78 and 95% (*n* = 64). Patients with estimated survival probability between 53 and 77% were in the third subgroup (*n* = 58), and patients with an estimated probability between 32 and 52% were in the fourth subgroup (*n* = 44). Patients with a survival probability less than 31% were in the fifth subgroup (*n* = 33). Kaplan-Meier curves were generated for each subgroup (Fig. 5b). All five different survival curves of the subgroups were clearly separated, with statistical significance (log-rank test; *p* < 0.001).

## Discussion

Although chondrosarcoma is the third most common primary malignant bone tumor, the spine is a relatively rare site that presents a challenge for surgery due to the potential for local recurrence [12–18]. Moreover, in chondrosarcoma, there has been no precise classification or subgrouping according to the prognosis due to its low incidence. Prediction models for survival or other prognostic factors have been explored using conventional statistical methods. Multivariable logistic regression has been one of the most widely used methods to identify risk factors of events, such as complications or death in cancer research [9, 10].

### ML techniques and SEER data in cancer research

Since cancer is associated with multidimensional factors, conventional linear statistical models have not shown reliable accuracy in predicting prognosis. To build non-linear statistical models, various ML techniques, including Bayesian networks (BNs), semi-supervised learning (SSL), support vector machines (SVMs), decision trees (DTs) and artificial neural networks (ANNs) have been widely applied to develop predictive models, resulting in accurate and powerful decision-making in cancer research. Zhou and Jiang [19] used DTs and ANNs in a survival analysis of patients with breast cancer. Delen et al. [20] compared logistic regression, DTs, and ANNs to predict breast cancer survivability. In addition, Endo et al. [21] compared seven methods in regard to prediction of 5-year survivability in diagnosed cases of breast cancer.

Due to the rarity of chondrosarcoma, only a few small case series of treatment outcomes have been reported over several decades at individual institutions [12, 13, 16, 17, 22–24]. The SEER database enables outcome analysis for a large number of patients based on attributes that are broadly classified as demographics (e.g., age, sex, location), therapeutic (e.g., surgical procedure, radiation therapy), and outcomes (e.g., survival period, cause of death). Previous investigations have analyzed survival period of patients from the SEER database based on demographics and prognostic determinants of primary osseous neoplasms of the spine. However, past works have not identified prognostic subgroups. In contrast, there is a great deal of published literature related to SEER data studies for other cancer types. Chen et al. and Fradkin used SEER database to analyze survival patterns in lung cancer [25, 26]. Fathy et al. studied colorectal cancer survival rate prediction in relation to the number of hidden layer in the ANN [27].

### Adoption of ANN model

ANNs, in particular, may allow accurate performance in the presence of unreliability, including incomplete data or measurement errors. Moreover, ANN could detect and recognize complex non-linear relationships between the variables [28]. In this regard, ANNs are expected to improve the predictive value of the retrospective data analysis. One of the earliest works in survival analysis using ANN was introduced by Faraggi and Simon [29], who used ANN as a basis for a nonlinear proportional hazard model. In a predictive model developed to evaluate the survival of women with breast cancer [30], the authors compared three classification models using the SEER database. They found that the calculated accuracy rates for SSL, SVM, and ANNs were 71, 51, and 65%, respectively. Our previous study also revealed that the patients with gastric cancer could be more accurately classified according to survival outcome by using SRN than classical TNM staging [31].

In this study, we developed a novel algorithm specific to survival analysis, and the results indicate it offers better performance compared to those in other studies using both a conventional statistical model and an ML model. Our RED model showed mean AUC of 0.85. In the calibration curve (Fig. 4b), our model has a tendency to over-estimate the survival of patients with poor prognosis and a tendency to under-estimate those with good prognosis, which is similar to a conventional logistic prediction model. We identified eight subgroups with an approximate predictive power of 32 attributes. Perioperative identification of these subgroups should help prognosticate survival as well as assist in guiding treatment modality for patients with spinal and pelvic chondrosarcoma.

Yet, the development of model base on ANNs is empirical, and some methodological issues remain to be unresolved. However, implementing statistics based on artificial intelligence could produce valuable information and clinical relevance including disease staging, patient’s prognosis, survival prediction and treatment decision making for physicians in clinical practice and should deserve further attention.

### Limitation and future direction

However, the present study has limitations. One of limitations is that variations in treatment strategies could not be accounted for, including radiation and chemotherapy and surgical strategies. Previous studies have shown that surgical techniques might have an impact on survival period, as chemotherapy regimens [18, 31–34]. Although chondrosarcoma examined in the study is resistant to chemotherapy, the SEER database does not contain detailed chemotherapy-related data. The SEER database also lacks information on surgical strategies including en-bloc and intralesional resection. Future multi-institutional studies may be warranted to determine the role of these variables as well that of advances in targeted radiotherapy and chemotherapy regimens, particularly in treating chondrosarcoma variants.

Secondly, artificial neural network has the ability to detect all possible interactions between variables. However it may act as a ‘black box’ and have limited ability to identify variables (or coefficient weights) used to create the models and possible causal relationships.

Lastly, the development and validation of the machines learning algorithms described in this study have not been externally validated in an independent physician-collected dataset or other registry such as the national Cancer Database (NCDB). As the model performance would be similar or much lower if an external dataset was used, the generalizability of the algorithm predictions remains to be determined, and future studies should seek to build on the findings presented here by examining prospectively collected registries.

## Conclusions

RED_SNN is a valid method to predict survival for spinal and pelvic chondrosarcoma, and it appears to be comparable to other methods. RED_SNN may offer the advantage of increased sensitivity for predicting longer or shorter OS.

## Supplementary information

**Additional file 1: Figure S1.** Experiments Networks **(A)** Final Network consists of Embedding Layer, LSTM Layer, 4 Fully Connected Layers. **(B)** Dropout0.3 Network adds Dropout Layer(0.3) between FC Layer and LSTM Layer on origin network. **(C)** Dropout0.5 Network adds Dropout Layer(0.5) between FC Layer and LSTM Layer on origin network. **(D)** Dropout0.7 Network adds Dropout Layer(0.7) between FC Layer and LSTM Layer on origin network. **(E)** Bignode network has randomly increased nodes in some Layers on the origin network.

**Additional file 2: Table S1.** 5-fold valid test accuracy about various networks.

## Data Availability

The datasets analyzed during the current study are available on the Surveillance, Epidemiology, and End Results (SEER) Program (www.seer.cancer.gov). We used samples from Incidence - SEER 18 Regs Custom Data (with additional treatment fields), Nov 2016 Sub (1973–2014 varying). In order to access the research data a formal request must be made to the SEER program.

## Abbreviations

ANNs: Artificial neural networks
AUCs: Areas under the ROC curves
BNs: Bayesian networks
c-index: Concordance index
DTs: Decision trees
ML: Machine learning
OS: Overall survival
RED_SNN: Risk Estimate Distance Survival Neural Network
ROC: Receiver operating characteristic
SEER: The Surveillance, Epidemiology, and End Results
SSL: Semi-supervised learning
SVMs: Support vector machines
