# Supplementary figures and images for: Novel prognostication of patients with spinal and pelvic chondrosarcoma using deep survival neural networks
Source: BMC Med Inform Decis Mak. 2020 Jan 6;20:3. doi: 10.1186/s12911-019-1008-4 (PMC6945432; doi:10.1186/s12911-019-1008-4)

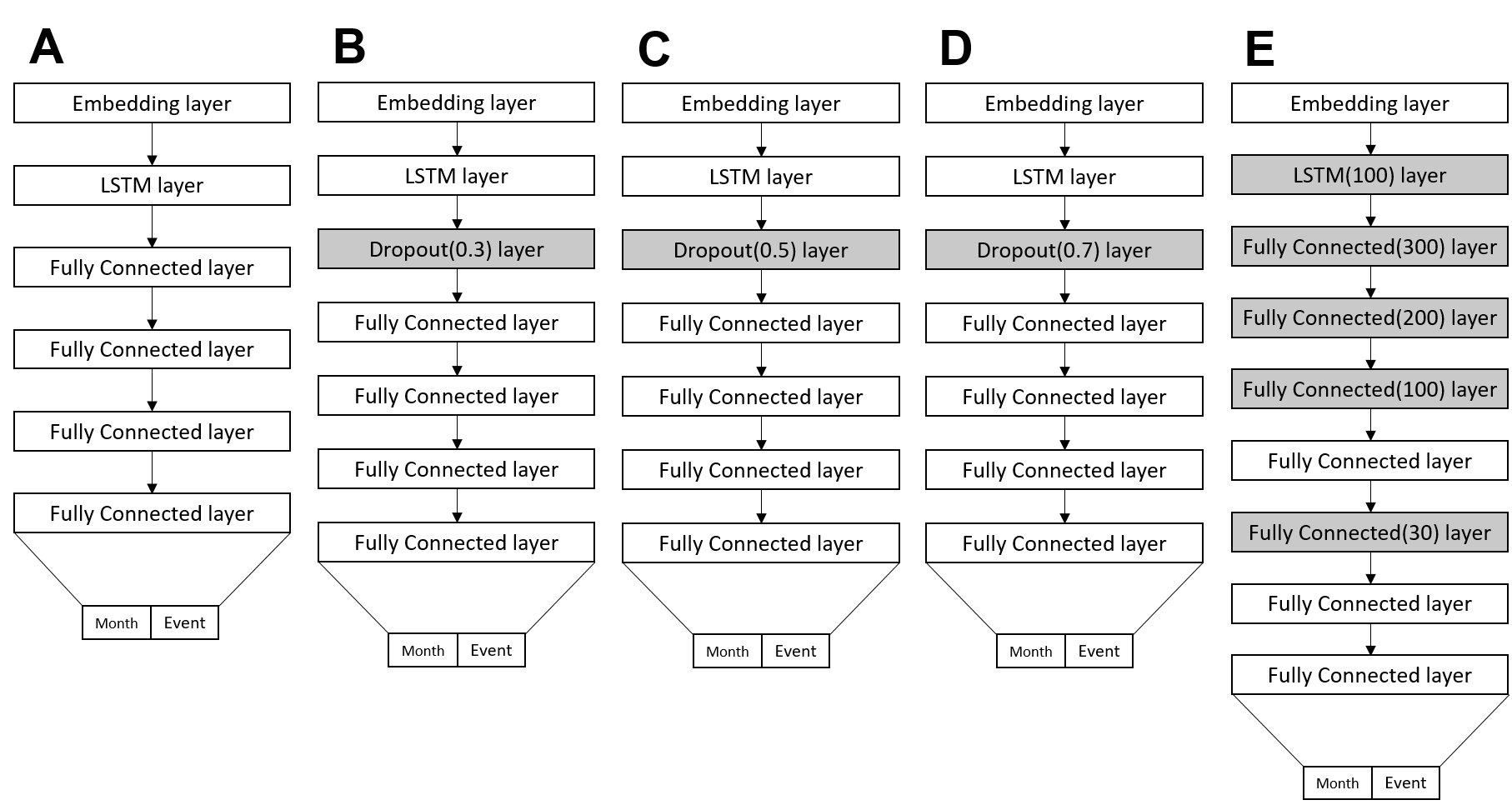

Supplement: Supplementary file 1 — Additional file 1: Figure S1. Experiments Networks (A) Final Network consists of Embedding Layer, LSTM Layer, 4 Fully Connected Layers. (B) Dropout0.3 Network adds Dropout Layer(0.3) between FC Layer and LSTM Layer on origin network. (C) Dropout0.5 Network adds Dropout Layer(0.5) between FC Layer and LSTM Layer on origin network. (D) Dropout0.7 Network adds Dropout Layer(0.7) between FC Layer and LSTM Layer on origin network. (E) Bignode network has randomly increased nodes in some Layers on the origin network. [file 12911_2019_1008_MOESM1_ESM.tif]
