# Supplementary material for: Novel prognostication of patients with spinal and pelvic chondrosarcoma using deep survival neural networks
Source: BMC Med Inform Decis Mak. 2020 Jan 6;20:3. doi: 10.1186/s12911-019-1008-4 (PMC6945432; doi:10.1186/s12911-019-1008-4)
Supplement: Supplementary file 2 — Additional file 2: Table S1. 5-fold valid test accuracy about various networks. [file 12911_2019_1008_MOESM2_ESM.docx]

Additional file 2: **Table S1.** 5-fold valid test accuracy about various networks

| networks |  | 1 year | 2 years | 3 years | 4 years | 5 years |
| --- | --- | --- | --- | --- | --- | --- |
| Final | mean | 0.795 | 0.793 | 0.792 | 0.789 | 0.799 |
|  | CI | 0.001 | 0.001 | 0.002 | 0.002 | 0.001 |
| Dropout0.3 | mean | 0.592 | 0.602 | 0.601 | 0.606 | 0.621 |
|  | CI | 0.003 | 0.003 | 0.003 | 0.003 | 0.003 |
| Dropout0.5 | mean | 0.689 | 0.680 | 0.682 | 0.683 | 0.691 |
|  | CI | 0.000 | 0.000 | 0.001 | 0.001 | 0.001 |
| Dropout0.7 | mean | 0.759 | 0.763 | 0.768 | 0.765 | 0.792 |
|  | CI | 0.001 | 0.001 | 0.001 | 0.001 | 0.001 |
| Bignode | mean | 0.619 | 0.619 | 0.620 | 0.618 | 0.623 |
|  | CI | 0.004 | 0.004 | 0.004 | 0.004 | 0.005 |

Final Network consists of Embedding Layer, LSTM Layer, 4 Fully Connected Layers.

Dropout0.3 Network adds Dropout Layer(0.3) between FC Layer and LSTM Layer on origin network.

Dropout0.5 Network adds Dropout Layer(0.5) between FC Layer and LSTM Layer on origin network.

Dropout0.7 Network adds Dropout Layer(0.7) between FC Layer and LSTM Layer on origin network.

The Bignode network has randomly increased nodes in some Layers on the origin network.

A simple structure for the network can be found in supplementary figure 1. Mean shows accuracy, CI shows a margin of error for accuracy.
